# Cytotoxicity Evaluation of Endodontic Pins on L929 Cell Line

**DOI:** 10.1155/2019/3469525

**Published:** 2019-10-30

**Authors:** Vincenza Cannella, Roberta Altomare, Gabriele Chiaramonte, Santina Di Bella, Francesco Mira, Laura Russotto, Patrizia Pisano, Annalisa Guercio

**Affiliations:** Istituto Zooprofilattico Sperimentale Della Sicilia “A. Mirri”, Via Gino Marinuzzi 3, 90129 Palermo, PA, Italy

## Abstract

**Objective:**

The aim of this study was to evaluate the cytotoxic potential of a type of endodontic pin on L929 cell line according to the UNI EN ISO 10993/2009 rule.

**Methods:**

L929 cells were used for the assays; extracts were prepared from three different-diameter endodontic pins, made of epoxy resin and fiberglass matrix and from Reference Materials (ZDEC, ZDBC, and HDP films). MTS assay was performed after 24 h, 48 h, and 72 h of exposure of L929 cells to pin and Reference Material extracts, 5% phenol solution, and control reagent. Cells cultured with different media containing extracts were monitored for up to 72 h and stained with haematoxylin/eosin.

**Results:**

Pins of different diameters had no cytotoxic effects on L929 cells at 24 h, 48 h, and 72 h (all values >70%). Cells cultured in medium containing pin extracts grew without any differences compared to the control cells.

**Conclusion:**

The endodontic pins tested showed no cytotoxic effects and did not induce changes in morphology for up to 72 h.

## 1. Introduction

Medical devices are designed for diagnostic, investigative, and treatment purposes and used in direct or indirect contact with the patient [1]. The Consensus Conference of the European Society for Biomaterials established that biomaterials are “non viable materials used in medical devices, intended to interact with biological systems” [2]. Dental pins, as medical devices, must satisfy the requirement of biocompatibility, that is, the ability to induce an appropriate biological response in a specific application, without causing damage or injury. This implies an interaction between the host environment, the material, and the function that it must perform [3]. In other words, “biocompatibility” means a dynamic process aimed to minimize any adverse reaction or rejection by the host [4]. In fact, since dental implants integrate with the bone, vital dentin, and dental pulp [5], the interaction of the material with the surrounding tissues could be responsible for many immunological alterations [6] or tissue reactions as inflammation and necrosis [7]. A type of endodontic pin, currently widely used, is composed of an epoxy resin and fiberglass matrix [8]. Fiberglass has high resistance and biocompatibility, showing extraordinary mechanical characteristics and a modulus of elasticity very similar to natural teeth [9]. However, marketing of any biomaterial or device, intended for human use or for a long-term contact, requires the assessment of the biological response [10]. For this purpose, the evaluation of biocompatibility is a crucial step to establish the safety of the material [11]. In recent years, in compliance with the European Directive 63/2010/EU [12], the interest of the scientific community towards the application of *in vitro* methods has grown due to the need of replacement or reducing the *in vivo* methods. Among the various methods, those based on the use of cell cultures are very widespread in toxicology because they are sensitive, reproducible, and certainly less expensive, compared to *in vivo* methods [13]. It is known that the interaction of cell cultures with a toxic compound or with its extract always induces a detectable biological response [14, 15]. Cytotoxicity tests estimate the possible alterations in basic cellular functions [16] that can be evaluated by analyzing cellular metabolism, structure, and proliferation rate or vitality [17, 18]. In this context, the UNI EN ISO 10993/2009 rule, describes how to perform the biological evaluation of medical devices. Particularly, part five gives specific guidelines for *in vitro* methods to evaluate the cytotoxicity of different types of materials. These methods consist on the incubation of cell cultures with a medical device, in order to evaluate the *in vitro* biological response. Tests can be performed on materials or on their extracts by direct or indirect contact, depending on the nature and shape of the material [19].

The aim of this work was to assess the cytotoxic potential of three different-diameter endodontic pins, used in dentistry for restoring devitalized teeth, through an *in vitro* method, alternative to animal use. The L929 cell lines were used for this purpose, according to the UNI EN ISO 10993/2009 rule [19].

## 2. Materials and Methods

### 2.1. Samples

Samples used were endodontic pins made of epoxy resin strengthened with fiberglass Richmond system (FiberSite post, Megadental Italia), available in three diameter sizes (2 mm, 4 mm, and 5 mm) matching up the mesiodistal diameters with the neck of all single-rooted teeth, in order to guarantee a perfect match between the abutment and root. ZDBC (zinc N,N-dibutyldithiocarbamate) and ZDEC (zinc diethyldithiocarbamate) films and 5% phenol solution were used as positive controls as indicated in the rule. These materials were chosen as positive controls due to their ability to induce a reproducible cytotoxic response. HDP (high-density polyethylene) films were used as negative controls because it was demonstrated that they do not induce any cytotoxicity [20]. ZDBC, ZDEC, and HDP films consisted 2 × 15 mm sheets (Food and Drug Safety Center, Hatano Research Institute).

### 2.2. Cell Culture

L929 cell line (murine fibroblast) was purchased from Cell Bank of National Reference Institute for Alternative Methods, Welfare and Care of Laboratory Animals (Istituto Zooprofilattico Sperimentale of Lombardia and Emilia Romagna). Cells were grown in culture flasks containing minimum essential medium (MEM, Sigma-Aldrich), supplemented with 10% fetal bovine serum (FBS, EuroClone), 1% antibiotic-antimytotic solution (Sigma-Aldrich), and 1% nonessential aminoacids (NEAA, EuroClone). Cells were maintained at +37°C in a humidified 5% CO_2_ atmosphere and monitored daily by using an inverted microscope. Subcultures were performed twice a week, when an 80% of confluence was observed.

### 2.3. Samples Preparation

Among the methods recommended by the UNI EN ISO 10993 regulation, the “extraction dilution method” was considered the most suitable for the shape and nature of the pin [20]. Before performing the extraction procedure, each sample was sterilized at +121°C for 20 minutes and then placed into a sterile, chemically inert, and closed flask, suitable for cell culture. The extraction procedure was carried out in MEM, supplemented with 10% FBS, 1% antibiotic-antimytotic solution, and 1% NEAA, at +37°C ± 1 for 72 h, by continuous agitation. ZDBC, ZDEC, and HDP sheets were extracted as a sample. The surface area of samples was used to determine the volume of the extraction vehicle needed [20]: 2 mm diameter pin was extracted in 5 ml of extraction medium; 4 mm diameter pin was extracted in 10 ml of extraction medium; 5 mm diameter pin was extracted in 12.5 ml of extraction medium. Extraction medium without sample (MEM with 10% FBS, 1% antibiotic-antimytotic solution, and 1% NEAA) was used as reagent control and treated as a sample.

### 2.4. Cytotoxicity Test on L929 Cell Line

Cells were seeded into 96-well culture plates at 1 × 10^4^ cells/ml ratio in MEM, supplemented with 10% FBS, 1% antibiotic-antimytotic solution, and 1% NEAA. Three 96-well culture plates for each sample of different diameter (2 mm, 4 mm, and 5 mm) were prepared and incubated at +37°C ± 1 in 5% CO_2_ for 24 h. After this time, culture media was replaced with 100 *μ*l of 100% concentrated sample and control extracts and a series of two-fold dilutions (from 1 : 2 to 1 : 32). The assay was carried out in triplicate. Moreover, intralaboratory assays were performed. Some wells were filled with MEM, supplemented with 10% FBS, 1% antibiotic-antimytotic solution, and 1% NEAA and used as control cells (negative control). All plates were incubated at +37 ± 1°C in a 5% CO_2_ atmosphere and examined microscopically after 24 h, 48 h, and 72 h of incubation in order to assess vitality and general morphology of cells. The vitality MTS assay was performed at the same time points. The MTS reagent (CellTiter 96 AQueous One Solution Cell Proliferation Assay—Promega) was directly put into each well containing samples and controls and incubated at+37 ± 1°C in a 5% CO_2_ for 4 h. During incubation, the metabolically active cells bioreduced MTS salt to formazan, which is soluble in the culture medium. This reaction occurs in the mitochondria through dehydrogenase enzymes. The absorbance was recorded at 490 nm with a 96-well plate reader. The absorbance is directly proportional to the number of living cells. All samples and controls were compared with negative control to calculate the percentage of vital cells, using the following equation:

(1) $$\begin{array}{c} \text{Viab }\%=100\times \frac{\text{O}.{\text{D}}_{490\text{e}}}{\text{O}.{\text{D}}_{490\text{b}}}, \end{array}$$

where O.D_490e_ is the mean value of the measured optical density of extracts and O.D_490b_ is the mean value of the measured optical density of the negative control. According to this equation, the O.D. of the cells treated with samples (pin extracts) and controls (positive, negative and reagent) was related to the O.D. of the negative control (cells without treatment) in order to estimate the percentage of viability. The lower viability % value indicates a higher cytotoxic potential. A sample is considered cytotoxic if the percentage vitality value is <70% and noncytotoxic if the percentage vitality value is >70%.

### 2.5. Hematoxylin/Eosin Staining on L929 Cell Line

Cells were seeded into 6-well culture plates at 1 × 10^5^ cells/ml ratio in seven different media: negative control (I), pin extract (II), ZDBC extract (III), ZDEC extract (IV), HDP extract (V), 5% phenol solution (VI), and reagent control (VII).

Cells were maintained at +37°C ± 1 in a humidified 5% CO_2_ atmosphere and monitored daily by using an inverted microscope for 72 h in order to evaluate cell morphology and monolayer integrity. After 72 h incubation, haematoxylin/eosin staining was performed. Briefly, media was removed from each well, and cells were washed with PBS and fixed in methanol; 1% haematoxylin (Sigma-Aldrich) solution was added, followed by PBS washing, and 1% eosin staining (Sigma-Aldrich). Cell morphology was evaluated by using an inverted microscope supplied with camera (Leica).

## 3. Results

**Figure 1:**
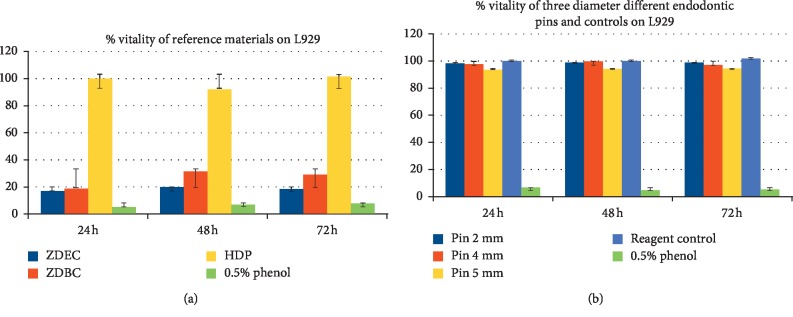
Effects of Reference Materials, pin extracts, and controls on L929 cells at 24 h 48 h, and 72 h of exposure. Data express the percentage of cell vitality after exposure to (a) ZDEC, ZDBC, and HDP extracts and phenol 0.5% and (b) the three different-diameter endodontic pin extracts.

The results of cell viability (MTS assay) showed that pins of different diameters had no cytotoxic effects on L929 cells at 24 h, 48 h, and 72 h (all values >70%).  Figure 1 shows results calculated using equation (1) for determining cell viability of L929 exposed to the different extracts.  Figure 1(a) shows the effect of Reference Materials and 0.5% phenol; Figure 1(b) shows the percentage of vitality of cells exposed to the three different-diameter pins and reagent control, compared with 0.5% phenol. Positive controls showed a cell viability <70% confirming their cytotoxic effect. 0.5% phenol solution induced a higher level of mortality (viability <9%), followed by ZDEC (viability <20%) and ZDBC (viability <32%). Diluted positive samples induced cytotoxicity until 1 : 4 dilution (data not shown). The HDP showed no cytotoxic effect (viability >70%). These results demonstrated the efficacy of the cellular system. Cells exposed to pin extracts showed a high level of vitality in all tests performed. No differences in cell viability were observed when the pin extracts were diluted.

**Figure 2:**
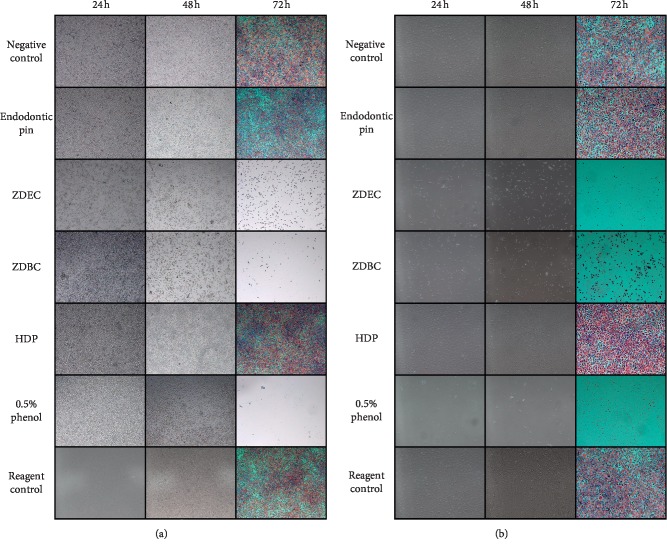
Morphologic evaluation of L929 cells exposed to different media. Observation conduced at 24 h (first column), 48 h (second column), and 72 h (third column) after haematoxylin/eosin staining. Lower (Figure 2(a)) and higher (Figure 2(b)) magnifications are shown. In the rows from top to bottom: negative control (medium I), pin extract (medium II), ZDEC (medium III), ZDBC (medium IV), HDP (medium V), phenol 0.5% (medium VI), and reagent control (medium VII).

Figure 2 shows the morphology of L929 when cultured in the seven different media for 24 h, 48 h, and 72 h. The 72 h column shows cells stained with hematoxylin/eosin. When cultured in medium II (endodontic pin), medium V (HDP), and medium VII (reagent control), cells grew without any differences compared with medium I (negative control) at different time points. In media III, IV, and VI (ZDEC, ZDBC, and 0.5% phenol, respectively), the cell monolayer was destroyed by the toxic effect of the substances present in the media and cell mortality increased during the observation time. No adherent cells were detected after the washing steps of the staining protocol.

## 4. Discussion

In this study, the potential cytotoxicity of three different-diameter endodontic pins made of epoxy resin and fiberglass matrix was evaluated in comparison with the effects of different Reference Materials on L929 cell line [20]. It is known that ZDEC, ZDBC, and 0.5% phenol solution have high cytotoxic effects on cell cultures. According to the UNI EN ISO 10993 regulation, these positive controls were used to determinate the level of their cytotoxicity on L929 cell line in order to compare the potential effect of endodontic material under examination. The viability test used was MTS assay, that is, a well-accepted cytotoxicity assay, due to its low cost, accuracy, speed, and reproducibility [6, 19]. Viable cells reduce MTS salt to formazan, which is soluble in the culture medium and detectable by spectrophotometer reading. This assay does not use radioisotope reagents, making it safe and easy to perform. In addition to MTS assay, L929 cells were directly exposed to the extract of pins and Reference Materials in order to observe how different media could influence cell growth and cell morphology.

Endodontic pins are highly used in dental clinical practice to restore the devitalized teeth. Pins made of epoxy resin and fiberglass are among the most used in dentistry due to their major elasticity and similarity to natural tissue. However, it is important that the application of a biomaterial is supported by scientific evidences [4] that demonstrate the safety of the product and the absence of biological risks for the patient. Both MTS assay and hematoxylin/eosin staining demonstrated that the three different-diameter pins have no cytotoxic effects on L929 cells. Cell viability and cell morphology resulted unaffected by the pin extracts in comparison with control cells at three different times of observation (24 h, 48 h, and 72 h), demonstrating that this biomaterial is safe.

Cell-based assays represent an increasing approach in toxicology, to reduce or replace the use of animal-based tests. *In vitro* methods based on cell culture are sensitive, reproducible, and low cost and have no ethical implications compared to *in vivo* assays. In fact, standardized and validated tests in cell culture could significantly limit the use of laboratory animals in biocompatibility tests. This study shows the suitability of an easy method to assess the biocompatibility of an endodontic device by evaluating viability and morphologic alterations of cell cultures.

## 5. Conclusions

The three endodontic pins tested were not cytotoxic and did not induce changes in morphology when evaluated on L929 cells in the incubation period of up to 72 h. As expected, Reference Materials and phenol solution used as positive controls induced relevant alterations in cell morphology and metabolism.

## Data Availability

The data of this study are available from the corresponding author upon request.

## References

[1] Lewandowska-Szumiel M. (1999). Alternative methods for assessing biocompatibility and function of implant materials. *Alternatives to Laboratory Animals*.

[2] Williams D. F. (1987). *Definitions in Biomaterials*.

[3] Pera P., Conserva E., Acquaviva A. (2004). Cytotoxicity analysis of composita materials for metal-free FPD: an in vitro investyigation. *Quintessenza Internazionale*.

[4] Catunda R.-Q., Vieira J. R. C., de Oliveira E. B., da Silva E. C., Brasil V. L., Perez D. C. (2017). Citotoxicity evaluation of three dental adhesives on vero cells in vitro. *Journal of Clinical and Experimental Dentistry*.

[5] Mantellini M. G., Botero T., Yaman P., Dennison J. B., Hanks C. T., Nör J. E. (2006). Adhesive resin and the hydrophilic monomer HEMA induce VEGF expression on dental pulp cells and macrophages. *Dental Materials*.

[6] Demirci M., Hiller K., Bosl C., Galler K., Schmalz G., Schweikl H. (2008). The induction of oxidative stress, cytotoxicity, and genotoxicity by dental adhesives. *Dental Materials*.

[7] Lodiene G., Morisbak E., Bruzell E., Ørstavik D. (2008). Toxicity evaluation of root canal sealers in vitro. *International Endodontic Journal*.

[8] Ferrari M., Vichi A., Garcia-Godoy F. (2000). Clinical evaluation of fiber-reinforced epoxy resin posts and cast post and cores. *American Journal of Dentistry*.

[9] Jukka M. Z., Matinlinna P. (2012). E-glass fiber reinforced composites in dental applications. *Silicon*.

[10] Escobar-García D. M., Aguirre-López E., Méndez-González V., Pozos-Guillén A. (2016). Cytotoxicity and initial biocompatibility of endodontic biomaterials (MTA and Biodentine™) used as root-end filling materials. *BioMed Research International*.

[11] Williams D. F. (2008). On the mechanisms of biocompatibility. *Biomaterials*.

[12] International Organization for Standardization (2009). *UNI EN ISO 10993:2009, “Biological Evaluation of Medical Devices”*.

[13] Hampshire V. A., Gilbert S. H. (2018). Refinement, reduction and replacement (3R) strategies in preclinical testing of medical devices. *Toxicologic Pathology*.

[14] Cetenovic B., Prokic B., Vasilijic S. (2017). Biocompatibility investigation of new endodontic materials based on nanosynthesized calcium silicates combined with different radiopacifiers. *Journal of Endodontics*.

[15] Karapınar-Kazandag M., Bayrak Ö. F., Yalvaç M. E. (2011). Cytotoxicity of 5 endodontic sealers on L929 cell line and human dental pulp cells. *International Endodontic Journal*.

[16] Li W., Zhou J., Xu Y. (2015). Study of the in vitro cytotoxicity testing of medical devices. *Biomedical Reports*.

[17] Asgary S., Shahabi S., Jafarzadeh T., Amini S., Kheirieh S. (2008). The properties of a new endodontic material. *Journal of Endodontics*.

[18] Osdemir K. G., Yilmaz H., Yilmaz S. (2009). In vitro evaluation of cytotoxicity of soft lining materials on L929 cells by MTT assay. *Journal of Biomedical Materials Research Part B: Applied Biomaterials*.

[19] International Organization for Standardization (2009). *UNI EN ISO 10993-5:2009, “Biological Evaluation of Medical Devices—Part 5: In Vitro Cytotoxicity Testing”*.

[20] International Organization for Standardization (2009). *UNI EN ISO 10993-12:2009, “Biological Evaluation of Medical Devices—Part 12: Preparation of Samples and Reference Materials”*.

